# Regulation of extrathymic Treg cell conversion by CD5

**DOI:** 10.18632/oncotarget.5809

**Published:** 2015-09-26

**Authors:** Jacob G. Henderson, Daniel Hawiger

**Affiliations:** Department of Molecular Microbiology and Immunology, Saint Louis University School of Medicine, St. Louis, MO, USA

**Keywords:** CD5, Treg cells, tolerance

The immune system must carefully regulate the balance between immunity and tolerance in order to prevent disease. Mechanisms of tolerance include crucial functions of thymically developed regulatory T (tTreg) cells as well as peripheral regulatory T (pTreg) cells that differentiate from T cells outside the thymus [1]. The development of pTreg cells is tightly regulated to promote tolerance to innocuous and self-antigens without compromising the ability of the immune system to remove offending pathogens. The autoimmune diseases can be caused by decreased functions of Treg cells but anti-tumor responses can instead be hampered by aberrant immune regulation. Many promising therapies revolve around correcting such imbalances of Treg cell functions. Therefore, the current intense research efforts to elucidate the mechanisms governing Treg cell differentiation could lead to new therapies to alleviate autoimmunity, inflammatory diseases and cancer [1, 2].

During thymic T cell development, T cells that respond strongly to self-antigens increase their CD5 expression to parallel the T cell receptor (TCR) signal strength and such T cells become CD5^hi^ [3]. Further, previous findings also revealed that some T cells that initially remained CD5^lo^ during thymic selection can still up regulate their CD5 expression outside the thymus in response to cognate self-antigens presented by peripheral tolerogenic dendritic cells (DCs) [4]. Consistent with CD5 expression correlating with TCR signal strength in thymus, expression of CD5 is also increased in regulatory T cells although CD5 is not required for the development of tTreg cells [5]. While the majority of CD5^hi^ cells in thymus do not develop into tTreg cells, the elevated CD5 expression persists in mature peripheral T cells to distinguish CD5^hi^ and CD5^lo^ T cells that responded with, respectively, high or low affinity to self-peptide(p)MHCs in thymus [6]. Despite the functions of CD5 as a negative regulator of TCR signaling, the CD5^hi^ T cells remain responsive to antigenic stimulation and are capable of forming effector T cells that are cross-reactive to self antigens thereby risking the development of anti-self responses [6]. The question then arises: how are such self-reactive T cells specifically instructed to convert into pTreg cells to help provide an antigen-specific tolerance?

**Figure 1:**
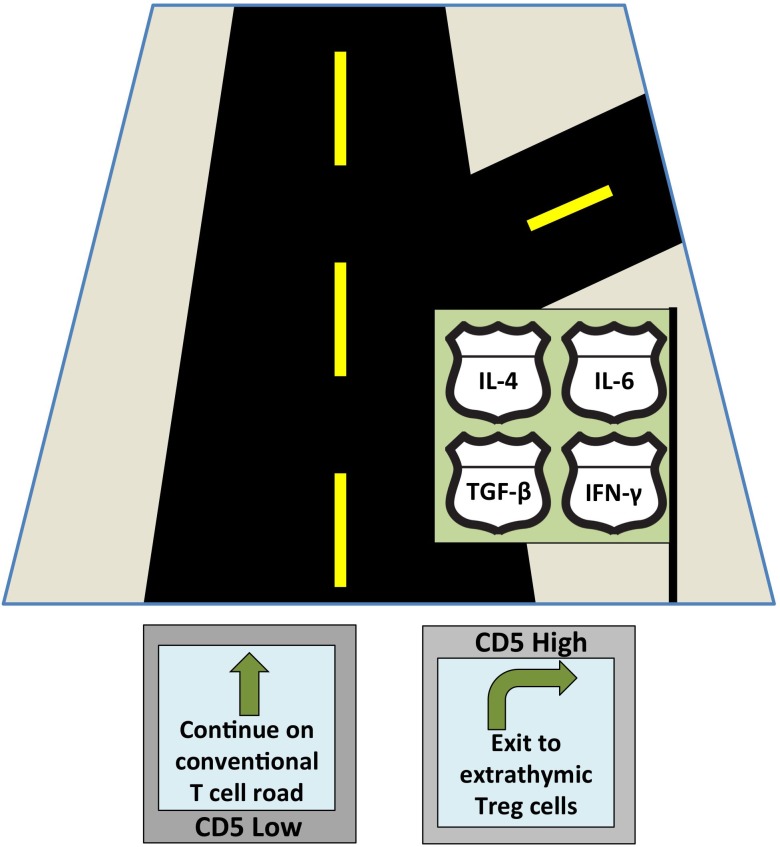
CD5 serves as a T cell “guidance system” navigating T cells towards Treg cell differentiation despite the presence of various cytokines that each signal a different developmental fate (represented by the confusing road signs).

Recently, we discovered a CD5-dependent mechanism to promote the conversion of self-reactive peripheral CD5^hi^ T cells into extrathymic Treg cells that block autoimmunity [7] and Figure 1. We found that CD5 promotes the conversion of such CD5^hi^ cells into Foxp3^+^ pTreg cells by blocking the mechanistic target of rapamycin (mTOR) activated in response to effector T cell-differentiating cytokines such as IL-4, IL-6, and IFN-γ [7]. These findings indicate that CD5^hi^ cells are less susceptible to the effector cytokine-mediated inhibition of Treg cell differentiation and therefore self-reactive CD5^hi^ T cells might preferentially convert into pTreg cells even during an on-going inflammatory process to alleviate autoimmunity. In contrast, CD5^lo^ T cells, which are not reactive to self or innocuous peripheral antigens but may be specific for foreign pathogens, have decreased conversion into Treg cells in the absence of CD5 functions [7]. Overall, CD5 regulates a selective extrathymic induction of Treg cells from T cells that have responded to high affinity self-pMHC in thymus or tolerizing antigens presented by tolerogenic DCs in the periphery, without compromising the general high plasticity of immune responses among the total T cell repertoire.

The role of CD5 in promoting tolerance to self-reactive antigens could also be exploited for future immune therapies. A selective increase of CD5 functions in autoaggressive T cells could enable careful targeting of tolerance thus avoiding general immunosuppression that is often associated with current immunomodulatory therapies. Additionally, tumor microenvironments are characterized by increased numbers of Treg cells that may prevent rejections of tumors despite the on-going pro-inflammatory process [2]. Since CD5 functions can enable the differentiation of pTreg cells despite the presence of effector T cell cytokines, it is attractive to speculate that tumors might manipulate the CD5 expression in responding T cells in a mechanism to skew T cell differentiation into pTreg cells, which then contribute to tumor survival. Disrupting such upregulation of CD5 in tumor-specific T cells could therefore prevent tumor cells from generating Treg cells necessary to block rejection of the tumor by the immune system without increasing the risk of autoaggressive immune responses. Undoubtedly, in order to develop such treatments, more work must be done to understand the mechanisms regulating expression and functions of CD5 in T cells. Doing so will also provide an insight into which pathways could become most promising targets for future immune therapies.
